# Genetic and ecological niche modeling of *Calydorea crocoides* (Iridaceae): an endemic species of Subtropical Highland Grasslands

**DOI:** 10.1590/1678-4685-GMB-2017-0078

**Published:** 2018

**Authors:** Juliana Lustosa Matos de Alencar, Eliane Kaltchuk-Santos, Juliana Fachinetto, Luana Olinda Tacuatiá, Eliana Regina Forni-Martins, Eudes Maria Stiehl-Alves, Tatiana Teixeira de Souza-Chies

**Affiliations:** 1 Programa de Pós-Graduação em Genética e Biologia Molecular, Universidade Federal do Rio Grande do Sul, Porto Alegre, RS, Brazil; 2 Departamento de Genética, Universidade Federal do Rio Grande do Sul, Porto Alegre, RS, Brazil; 3 Departamento de Biologia Vegetal, Universidade Estadual de Campinas (Unicamp), Campinas, SP, Brazil; 4 Programa de Pós-Graduação em Botânica, Universidade Federal do Rio Grande do Sul, Porto Alegre, RS, Brazil; 5 Departamento de Botânica, Universidade Federal do Rio Grande do Sul, Porto Alegre, RS, Brazil

**Keywords:** Genetic differentiation, ISSR markers, pollen viability, cytogenetic analyses, restricted distributed species

## Abstract

Evolutionary analyses have been widely used for evaluation of genetic diversity of natural populations and correlate these data to the fitness of the species, especially in the case of threatened species. *Calydorea crocoides* occurs in a restricted area at altitudes from 800 to 1500 m in southern Brazil and is considered endangered. A study assessing genetic diversity, cytogenetic features and ecological niche was performed aiming to characterize *C. crocoides* by multidisciplinary approaches. Molecular data highlighted that most of the total variation (76%; *p* < 0.001) was found within populations and the parameters of genetic diversity were high at the species level (*PPB* = 98.97%; *I* = 0.4319; *h* = 0.2821). Gene flow (*Nm*) was estimated in 0.97 individuals per generation. Cytogenetically, *C. crocoides* presents a bimodal karyotype and low asymmetry. DAPI banding pattern was uniform, but the CMA-signal evidenced a pericentric inversion in the population ESC688. The species presents high pollen viability and two different morphologies of pollen grains. Our data showed high levels of polymorphism maintained in this species that could ensure conservationist practices in which the main goal is to preserve the evolutionary potential of the species through the maintenance of genetic diversity.

## Introduction

Natural populations can be affected by several relevant factors such as mating system, population density, microhabitat selection, besides historical events, as disturbance and colonization. Thus, investigating these factors is of interest for population geneticists (Roser *et al.*, 2017). All these factors and others influence the distribution of the genetic diversity at the species level. The study of such factors and their relation with the genetic variability is essential for comprehension of the evolutionary scenario. Studies based on the estimation of genetic diversity are also essential for monitoring natural populations and to suggest conservation strategies concerning species of restricted distribution. Genetic data have been used to determine the current state of diversity of a species, the genetic structure and genetic differentiation between populations and thus, serve to establish the most suitable management strategy (Toro and Caballero, 2005; Frankham *et al.*, 2010; Furlan *et al.*, 2012; Kahilainena *et al.*, 2014; Pérez *et al.*, 2015).

Conservation biology approaches have been used to better understand evolutionary processes at all levels of biodiversity. For this, it is necessary to consider not only population numbers and sizes but also genetic diversity at the population level (Olivieri *et al.*, 2016). Preserving genetic diversity among populations, in particular those linked to local adaptations, implies understanding the evolutionary forces that may have shaped this diversity (Toro and Caballero, 2005; Szczecinska *et al.*, 2016). Species with restricted distribution can present low (Hannan and Orick, 2000; Szczecinska *et al.*, 2016) or high genetic diversity (Lorenz-Lemke *et al.*, 2006, 2010; Sede *et al.*, 2012; Barros *et al.*, 2015; Turchetto *et al.*, 2016), and genetic variability is the feedstock for evolution.

The fast pace of technological advances in the genomic and postgenomic eras has been greatly important for fostering knowledge on plant biology, but, when such data are not available, classical genetic analyses are still needed as a first step towards the description of biodiversity, especially for wild species (Pérez *et al.*, 2015). Studies concerning the estimation of genetic diversity have been done for several species of Iridaceae (Hannan and Orick, 2000; Wróblewska *et al.*, 2003; Sik *et al.*, 2008; Wang *et al.*, 2009; Tacuatiá *et al.*, 2012a,b; Stiehl-Alves *et al.*, 2016, 2017). Many molecular markers can be used as tools for population genetics studies in plants. Among these, ISSR markers “Inter-Simple Sequence Repeats” are particularly advantageous for analysis of unknown genomes when there is a lack of genomic information for the taxon studied (Zietkiewicz *et al.*, 1994; Garriga *et al.*, 2013). This is especially of interest when native, rare or endemic species are in focus, as in the present work. Notwithstanding, understanding species evolution requires the employment of multidisciplinary approaches to ensure confidence in established evolutionary hypotheses. Thus, cytogenetic analyses may contribute to the comprehension and characterization of karyotypic and genetic variability, besides providing information on genome variation and evolution (Storme and Mason, 2014). Iridaceae are cytologically well known (Goldblatt, 1982; Goldblatt and Takei, 1997; Moraes *et al.*, 2015), and a phylogenetic approach was recently used to analyze chromosome evolutionary trends, with emphasis on South American species of Iridaceae (Moraes *et al.*, 2015). The results of this study emphasized large variability in cytogenetic features among South American Iridaceae and suggest that chromosome evolution is important to species diversification. Similarly, establishing range limits for species polymorphism likewise are a challenge, because most species’ ranges are not limited by obvious dispersal barriers. Ecological niche modelling may help to identify the preferences of a given species, based on its known distribution (Aguilar and Lado, 2012). Species of restricted distribution possess a unique set of conditions, habitat availability and environmental preferences, under which their entire evolutionary process occurred (Aguilar and Lado, 2012). Combining genetic diversity, evolutionary aspects, and ecological niche modeling analysis can represent valuable tools for understanding the evolutionary dynamics of species, as well as assisting in the conservation of endangered species (Sohn *et al.*, 2013).

*Calydorea crocoides* Ravenna (Iridaceae: Iridoideae: Tigridieae) is a species found in grasslands, fields with herbaceous slopes of hills, at high altitudes in Campos de Cima da Serra, and has restricted distributed in this region (Eggers, 2015), although,collection data indicate its occurrence also in the state Minas Gerais (Supplementary material Table S1). The most recent publication listing the endangered flora of the state Rio Grande do Sul classified *C. crocoides* as “endangered” [EN, B1ab(iii)], considering as criteria its geographic range and continuous decline of the extension and quality of its habitat (Rio Grande do Sul, 2014). The species is also assessed as “Least Concern” (LC) in the Red List of Threatened Species of the International Union for Conservation of Nature –IUCN (Contu, 2013). Although this classification suggests that populations are stable and have no real known threats, the author emphasizes that accurate population data and ecological studies are lacking for *C. crocoides*. Thus, the use of multidisciplinary approaches to characterize this species is justified for providing information to review its threat status.

Considering that *C. crocoides* is a species of restricted distribution, could one expect a lower genetic diversity than for species displaying a wide geographic distribution? Taking into account its distribution pattern, the genetic differentiation among populations would be high or low? And how is the fertility of the populations? The present study aimed to investigate the genetic diversity and differentiation of natural populations of *C. crocoides,* having as specific aims to (a) evaluate the levels of genetic diversity in natural populations, (b) determine the degree of differentiation among populations, (c) characterize them cytogenetically, (d) analyze pollen viability of the populations, and (e) identify the potential distribution focused on suitable habitats for the species. These results are useful for proposing a threat status review for *C. crocoides* in the IUCN Red List, as well as to know if it is necessary to initiate certain *in situ* conservation strategies.

## Material and Methods

### Plant material

**Figure 1:**
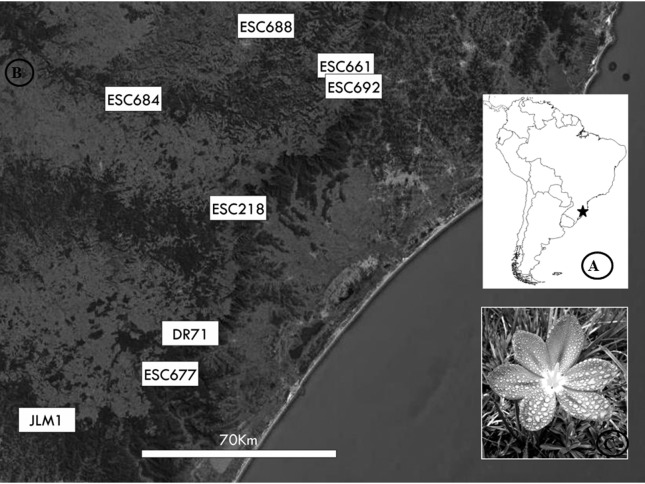
Locations of the studied sampling sites of *Calydorea crocoides*. A) map of South America; B) sampling sites in southern Brazil; C) *Calydorea crocoides*. Photograph: J. Fachinetto.

Specimens of *Calydorea crocoides* were collected at 10 sites located in the Subtropical Highland Grasslands of southern Brazil (Figure 1; Table 1). Although this area is extremely important because of the great diversity of species (296 endemic plant taxa were recently recorded), it is neglected by conservation policies (Overbeck *et al.*, 2007; Iganci *et al.*, 2011; Barros *et al.*, 2015). The samples were used for molecular and cytogenetic analyses. Voucher specimens were deposited in the ICN Herbarium, Instituto de Biociências, Universidade Federal do Rio Grande do Sul, Porto Alegre, Brazil.

**Table 1:** Sampling data of the populations analyzed of *Calydorea crocoides* in southern Brazil.

Population	Location	Latitude (°S)	Longitude (°W)	Altitude (m)	*Voucher*
ESC 661	Bom Jardim, SC	28°21’41.0”	49°35’20.0”	1360	173569 ICN
ESC 692	Bom Jardim, SC	28°23’08.3”	49°33’18.6”	1450	Eggers & Souza-Chies 692
ESC 688	São Joaquim, SC	28°13’43.2”	49°50’51.3”	1481	Eggers & Souza-Chies 688
ESC 218	São José dos Ausentes, RS	28°48’09.2”	49°57’0.35”	1350	173567 ICN
ESC 677	Tainhas/RS	29°18’59.8”	50°09’59.5”	946	Eggers & Souza-Chies 677
ESC 684	Bom Jesus, RS	28°28’53.9”	50°19’49.0”	1073	Eggers & Souza-Chies 684
JLM-1	São Francisco de Paula, RS	29°27’21.9”	50°36’28.3”	897	173415 ICN
DR 71	Cambará do Sul, RS	29°10’40.4”	50°06’15.2”	954	173573 ICN
ETLP807^a^	São Francisco de Paula, RS	29° 26’ 51.3"	50° 36’ 18.4"	908	180202 ICN
ETLP812^a^	São Francisco de Paula, RS	29° 27’ 16.6"	50° 36’ 25.5"	915	180203 ICN

ESC indicates the collectors Eggers and Souza-Chies, followed by the number of the collection, DR indicate Dal Ri, JLM collector Juliana Lustosa Matos, ETLP indicates the collectors Eggers *et al*.

(^a^) indicates the populations not included in molecular analyses.

### Molecular analysis

Leaves dried on silica gel from 25–30 specimen per population were used for total genomic DNA extraction following the modified protocol of Doyle and Doyle (1987). Thirty-one ISSR primers were tested with respect to repeatability, band sharpness of the fragments, and percentage of amplified specimens. Seven of these primers were selected for DNA amplification for all sampled specimen (Table 2). PCR assays were set up in a volume of 25 μL and the conditions optimized for each primer set were as follows: 3 μl of DNA (10 ng/μL), 2.5 μL buffer 10x, 0.8-1 μLof dNTPs (10 mM), 1.2-2.0 μL MgCl_2_ (50 mM), 1 μL primer (10 pmol/μL), 0.2 μL *Taq* DNA polymerase (5 U/μL) (Invitrogen, Carlsbad, USA). A negative control was added and run by replacing template DNA with H_2_O. Amplifications were performed using a Biocycler MJ96G thermocycler (Applied Biosystems Brazil Ltda., São Paulo, SP) with an initial denaturation step of 5 min at 94 °C, followed by 40 cycles of 1 min at 94 °C, 45 s at 48 °C, 2 min at 72 °C, and 5 min final extension step at 72 °C. PCR products were analyzed on 1.5% agarose gels and stained with GelRed (Amicon Corp., Lexington, USA). The size of the fragments was estimated using Ladder 100 bp (Ludwig Biotec, Porto Alegre, RS). Similar to the approach employed by Stiehl-Alves *et al.* (2016, 2017) and by Ng and Tan (2015), reproducibility of ISSR banding patterns was tested with three repeated amplifications of five samples per population. These did not show differences in the pattern of amplified DNA fragments.

**Table 2:** ISSR primers, size range (bp) of the amplified fragments, number of loci (*A*), and polymorphism information content (*PIC*) for *Calydorea crocoides.*

Primer	Code primer	Size range (bp)	A	*PIC*
(AG)_8_YC	F03	250 –2080	17	0.26
(GA)_8_YC	F04	250 –2080	17	0.23
(GTGC)_4_	F12	400 –1600	11	0.34
(GACA)_4_	F11	200 –2080	18	0.24
(GA)_8_C	SV8	500 –2080	14	0.23
A(TG)_8_	SV4	700 –1400	7	0.25
(AG)_8_C	SP2	400 –2080	13	0.27
Mean value	–	–	13.86	0.26

The amplified fragments were scored for band presence (1) or absence (0), and a binary qualitative data matrix was produced. Genetic parameters, including the percentage of polymorphic bands (*PPB*), observed number of alleles (*N*a), effective number of alleles (*N*e), the Shannon’s information index (*I*) (Lewontin, 1972), Nei’s gene diversity (*h*) (Nei, 1973) and gene flow (*N*_*m*_) (McDermott and McDonald, 1993) were calculated using POPGENE version 1.31 software (Yeh *et al*., 1999). The procedure implemented in HICKORY version 1.1 (Holsinger *et al.*, 2002) was also employed to infer estimates of within population genetic diversity (*hs*), assuming a full model, here defined as average panmictic heterozygosity. To assess the genetic similarity of individuals across populations, we computed the Jaccard similarity coefficients for all pairs of individuals, and an UPGMA dendrogram was generated using the program NTSYSpc version 2.1N (Rohlf, 2001). The dendrogram was edited in MEGA 5.0 (Tamura *et al.*, 2011). A hierarchical analysis of molecular variance AMOVA (Excoffier *et al.*, 1992) was obtained with the GenAlEx version 6 program (Peakall and Smouse, 2006) to determine the variance components, their significance levels, and Φ_ST_ (a statistic analogue to *F*_ST_). Total variance was partitioned within populations or among population levels. Pairwise Nei’s genetic identity was also estimated in GenAlEx to assess the similarity of the sampled populations. The number of permutations for significance testing was set at 999. A Mantel test with 999 permutations was also computed using GenAlEx 6.

A Bayesian analysis of population structure was performed on the entire data set using the program STRUCTURE version 2.3.1 (Falush *et al.*, 2007) to detect population structure and estimate the likely number of populations (*K*) in a sample. The most likely number of populations (*K*) was estimated under the admixture model and correlated allele frequencies, with no prior information on population origin. The program was run for 50,000 iterations, after a burn-in length of 1,000,000 iterations, to test population subdivision from *K* = 1 to *K* =16, and thereby checking for any possible subdivision. Fifty runs were carried out for each *K* to quantify variation in likelihood, as a means of checking whether different runs could produce different likelihood values. Individual and average admixture proportions (*Q*) for each population in each genetic cluster found by the program were recorded for the model. As an aid in identifying the number of clusters of individuals (*K*), the results generated by STRUCTURE were subsequently analyzed by the program STRUCTURE HARVESTER version 0.6.7 (Earl and von Holdt, 2012) according to the method of Evanno *et al.* (2005). In order to test clusters inferred by STRUCTURE, we also used STRUCTURAMA 2.0 (Huelsenbeck *et al.*, 2011). Using STRUCTURAMA, the number of populations was considered a random variable, and two different analyses were run, one considering no admixture between populations, and the other considering potential admixture. This analysis was based on runs of 100,000 Markov chain Monte Carlo (MCMC) cycles and a burn-in length of 10,000 iterations.

### Cytologic analyses

#### Karyotype analysis and chromosome banding

Mitotic analyses were performed with plants from five populations (ESC684, ESC688, ESC692, ETLP807, ETLP812). Root meristems were collected from bulbs of plants in pots and pre-treated with 2 mM 8-hydroxyquinoline solution for 24 h at 4 °C and subsequently fixed in fresh 3:1 (v/v) ethanol-acetic acid solution. Slides were prepared based on modified protocols of a classical squash technique (Schwarzacher and Leitch, 1994) or of a protoplast suspension (Murata, 1983), as described by Tacuatiá *et al.* (2017). Other methodological changes were: the enzyme mixture contained 1% (w/v) macerozyme R-10 (Serva, Heidelberg), 2% (w/v) cellulase RS (Serva), and 20% (w/v) pectinase (Sigma Aldrich Co., Steinheim); the digestion time was 12-18 min depending on root size, and slides were mounted with a medium composed of glycerol/McIlvaine buffer (pH 7.0) 1:1 (v/v), plus 5 mM MgCl_2_.

Staining of chromosomes was performed according to the protocol of Schweizer (1980), using chromomycin A_3_ (CMA_3_) and 4’,6-diamidino-2-phenylindole (DAPI) with some modifications: chromomycin staining was done for 1.5 h, followed by DAPI for 45 min. Segments of the chromosomes displaying enhanced (^+^), neutral (^°^) or reduced (^–^) fluorescence are denoted in the text following the fluorochrome name or fluorochrome combination. Metaphase chromosomes were observed and photographed with a fluorescence microscope Olympus BX51 (Olympus Co., Tokyo,) coupled with a DP72 digital camera and an imaging software DP2-BSW (Olympus). Metaphase plates with the best chromosome spreading and similar condensation levels were chosen. Measurements and karyotype analysis of 28 cells were made with the software KaryoType version 2.0 (Altinordu *et al.*, 2016).

Chromosomes were classified based on arm ratio, as proposed by Levan *et al.* (1964). Besides somatic (2*n*) and basic (*x*) chromosome numbers, karyotype description included three karyological parameters, which properly adress karyotype assymetry according to Peruzzi and Eroglu (2013): THL (total length of haploid chromosome set), M_CA_ (Mean Centromeric Asymmetry for an intrachromosomal asymmetry estimation; Peruzzi and Eroglu, 2013), and CV_CL_ (Coefficient of Variation of Chromosome Length, measuring the interchromosomal asymmetry; Paszko, 2006). Karyotype asymmetry was also evaluated based on the Stebbins’ method (Stebbins, 1971). Since some measures suggested possible different means between homolog chromosomes in relation to total length –short arm (S) plus long arm (L) -, statistical analysis was conducted using the Stats Package of R software version 3.3.2 (R Core Team, 2016) to verify possible heteromorphisms. First, normality and homoscedasticity of data were verified using shapiro.test (Shapiro-Wilk test) and var.test (F test) functions to determine whether parametric test could be used. Since the pair of chromosomes were not in accordance with one or both assumptions, the wilcox.test function (Wilcoxon–Mann–Whitney test) was applied to verify differences between means.

#### Morphology and stainability of pollen grains

The material used for pollen analysis consisted of inflorescences of 5-10 individuals per population (ESC 692, ESC 688 and ESC 677), which were collected in the field and fixed in an ethanol: acetic acid solution (3:1) for 12-24 h at room temperature. Slides were prepared following the method of Alexander (1980), wherein the empty unviable pollen grains are stained in green and viable pollen grains in purple. To determine pollen viability, samples of 500 pollen grains per flower from at least seven individuals per population were analyzed. Measurements of the polar axis (*P*) and equatorial diameter (*E*) of 20 pollen grains per slide were performed to determine the pollen morphology according to Erdtman (1971). Pollen grains characters were submitted to analysis of variance (ANOVA) and averages were compared by the Tukey test at 5% using BioEstat 5.0 software (Ayres *et al.*, 2007).

### Mapping and niche modelling

Data collection of all populations of *Calydorea crocoides* found between the years 2004 to 2016 were used to construct a distribution map for the species (Table S1). The map was constructed using the program DIVA-GIS version 7.5 (Hijmans *et al.*, 2005). This included a survey of the protected areas within the occurrence area of the species, and these were placed on the map. The potential distribution of suitable habitats for *C. crocoides* was modeled with the Bioclim algorithm based on the 19 bioclimatic variables of the Worldclim database version 2.0 (http://www.worldclim.org), at a resolution of five minutes per pixel. These variables come from monthly values of temperature and precipitation, represented by means, seasonality, and extreme temperature and precipitation conditions throughout the year, all these being widely used in ecological niche modeling studies (Hijmans *et al.*, 2005). A model was constructed combining the bioclimatic variables and known occurrence data for the species (Table S1) using DIVA-GIS version 7.5 (Hijmans *et al.*, 2005). From this model generated, MaxEnt (version 3.3.3) was used to produce a map of potential geographical distribution (Phillips *et al.*, 2006). MaxEnt is a robust method and has been shown to perform well with limited sample sizes in comparison to alternative approaches (e.g., Hernandez *et al.*, 2006). MaxEnt uses environmental data from occurrence records and background samples, in order to estimate the ratio between these. It works by making an estimate of distribution values for the presence records that are consistent with occurrence data, and chooses the distribution that is closest to the distribution of values for the background. Minimizing distance from background assumes that the species occupies environmental conditions proportional to their availability in the landscape. Distance from the background is considered as the relative entropy of occurrence data with respect to background (Elith *et al.*, 2011). The results obtained are shown as environmental suitability, with values ranging from 0–1. Again lowest presence threshold (LPT) and jackknife analyses were applied on MaxEnt version 3.3.3. Omission/commission errors and true skill statistic (TSS) were calculated from field points using the same program.

## Results

### Genetic diversity

In this study, genetic diversity was examined for *Calydorea crocoides* based on ISSR fingerprinting. Seven primers were selected to assay a total of 235 individuals from eight populations (Table 2). The seven primers produced a total of 97 fragments that were clearly identifiable. The size of the amplified products ranged from 200 to 2080 bp and the number of amplified fragments for each primer from seven [A(TG)_8_] to 18 [(GACA)_4_] with an average of 13.86 per primer (Table 2). No specific fragment was detected in the analyzed populations. The polymorphism information content (PIC) ranged from 0.23 to 0.34, with an average of 0.26 (Table 2). At the species level, the effective number of alleles was *N*e = 1.4724, Shannon’s index was *I* = 0.4319, percentage of polymorphic bands was *PPB =* 98.97%, Nei’s gene diversity was *h* = 0.2821, and Bayesian genetic diversity was *hs* = 0.1950 (Table 3). At the population level, *N*e = 1.3268 (range from 1.2280 to 1.3986), *I* = 0.2771 (range from 0.1970 to 0.3276) and *PPB* = 51.16 (range from 36.08% to 61.86%). Assuming Hardy-Weinberg equilibrium, the average Nei’s gene diversity was *h* = 0.1873 and varied between 0.1331 and 0.2233 (Table 3). Bayesian genetic diversity was *hs* = 0.1464, with a range from 0.1418 to 0.2238, similar to that obtained for Nei’s index.

**Table 3:** Genetic diversity index within populations of *Calydorea crocoides*.

Populations	*N*_a_	*N*_e_	*H*	*I*	*PPB*	*hs*
ESC 661	1.422	1.228	0.136	0.207	42.27	0.141
ESC 218	1.360	1.231	0.133	0.197	36.08	0.150
ESC 677	1.567	1.380	0.217	0.319	56.70	0.220
ESC 684	1.577	1.398	0.223	0.326	57.73	0.221
ESC 688	1.494	1.298	0.176	0.263	49.48	0.200
ESC 692	1.587	1.396	0.223	0.327	58.76	0.223
JLM-1	1.618	1.378	0.214	0.318	61.86	0.215
DR- 71	1.463	1.301	0.174	0.257	46.39	0.185
Mean value	1.511	1.326	0.187	0.277	51.16	0.146
Species level	1.989	1.472	0.282	0.431	98.97	0.195

*N*_a_: observed number of alleles; *N*_e_: effective number of alleles; *H*: Nei’s (1973) gene diversity; *I*: Shannon’s information index; *PPB*: percentage of polymorphic bands*, hs*: Bayesian genetic diversity.

### Population genetic structure

The results provided by the analysis of molecular variance (AMOVA) showed significant genetic variation (24%; *p* < 0.001) among populations, and the rest of total genetic variance (76%; *p* < 0.001) was attributable to within-populations diversity (Table 4). The estimated Φ_ST_ (0.24) indicated genetic differentiation among populations. Average gene flow was *N*_*m*_ = 0.87 individuals per generation. A pairwise matrix comparing *N*_*m*_ and Φ_ST_ between populations was estimated and emphasized that the populations ESC218 and ESC661 showed greater genetic differentiation; pairwise gene flow ranged from 1.01 to 2.82 (Table 5). The relationship between genetic distance and corresponding geographic distance among populations tested by the Mantel test showed that there is no significant correlation between geographic and genetic distances (*r* = 0.299, *p*=0.089).

**Table 4:** Analysis of molecular variance (AMOVA) within and among populations.

Source of variance	DF	SS	MS	%	Φ_ST_	^a^ *P*-value
Among Populations	7	1650.17	235.74	24	0.24	< 0.001
Within populations	227	4912.45	21.64	76		< 0.001

a Significance tests after 1,000 permutations.

DF: degree of freedom. SS: sum of squares. MS: expected mean squares. %: Percentage of the variance.

**Table 5:** Pairwise Φ_ST_ (below diagonal) and gene flow (*N*_m_, above diagonal) indices estimated for populations of *Calydorea crocoides*.

Populations	ESC661	ESC218	ESC677	ESC684	ESC688	ESC692	JLM-1	DR-71
ESC661	-	1.15	1.46	1.49	1.61	1.32	1.47	1.08
ESC218	0.41	-	1.10	1.46	1.51	1.26	1.20	1.01
ESC677	0.42	0.41	-	2.57	2.40	2.82	2.21	2.11
ESC684	0.36	0.33	0.11	-	2.08	2.24	1.92	2.02
ESC688	0.31	0.31	0.15	0.14	-	2.34	2.13	2.02
ESC692	0.36	0.34	0.10	0.13	0.11	-	2.60	2.22
JLM-1	0.36	0.37	0.17	0.20	0.14	0.12	-	1.92
DR-71	0.41	0.40	0.07	0.14	0.13	0.08	0.17	-

**Figure 2:**
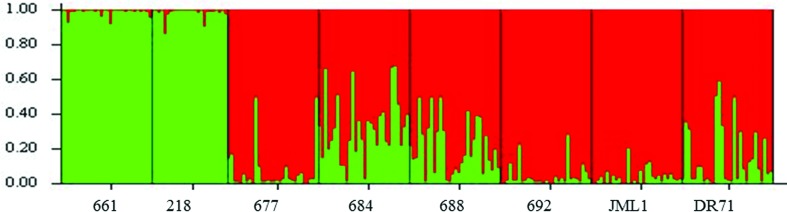
Bayesian admixture proportions (*Q*) of individuals of *Calydorea crocoides* para *K* = 2 identified in the STRUCTURE, shown in different colors. The most likely number of populations (*K*) was estimated with the admixture model and correlated allele frequencies, with no prior information regarding population origin.

**Figure 3:**
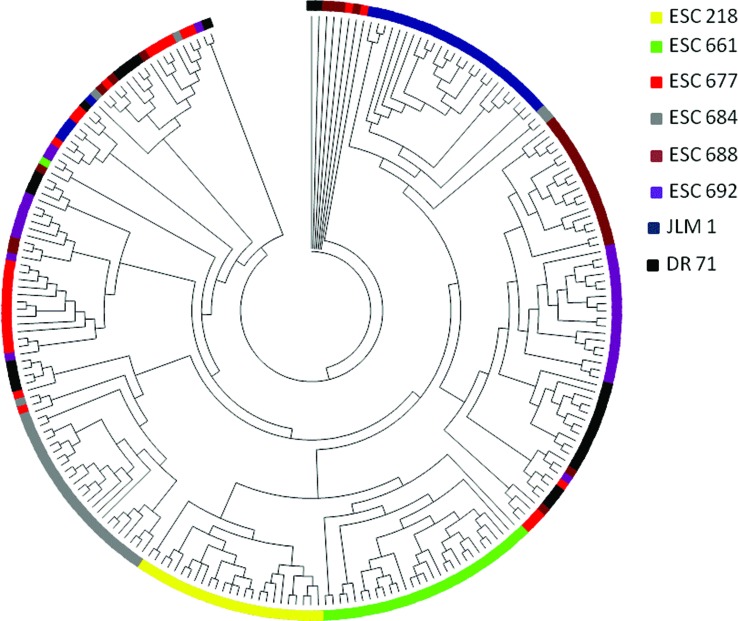
Unweighted pair-group method arithmetic average (UPGMA) dendrogram based on Jaccard’s index showing genetic similarity among all pairs of individuals of *Calydorea crocoides*.

According to Evanno’s method, the STRUCTURE analysis indicated *K* = 2 as the most likely number of genetic clusters to the surveyed populations of *C. crocoides*, a group composed by the populations ESC661 and ESC218, the other populations grouped in a second genetic cluster (Figure 2). These genetic pools were consistent with the UPGMA dendrograms estimated with the Jaccard index (Figure 3). However, when considering the populations as a random variable (STRUCTURAMA analysis), only one cluster was inferred for the entire dataset, considering no admixture (*p* = 0.89) or populations potentially admixture (*p* = 0.97). In accordance with the STRUCTURAMA results, the pairwise genetic identity of sampled populations was close to 1, showing a high degree of similarity among populations (Table S2). Pairwise Nei’s genetic identity range from 0.769 (population ESC692 versus DR71) to 0.905 (ESC677 versus ESC688).

### Karyotype analysis and chromosome banding

**Figure 4:**
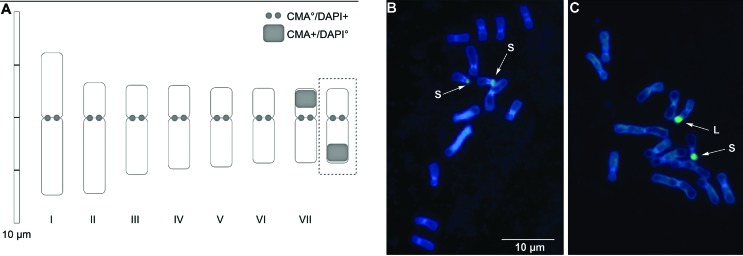
Idiogram and fluorescent chromosome banding with CMA/DAPI on somatic chromosomes of *Calydorea crocoides*. A) Idiogram showing seven chromosome pairs. Inside the dotted box, a chromosome variant found of pair seven (ESC688), with an inversion of the GC-rich DNA region (CMA^+^/DAPI°). B) and C) Metaphase plates with fluorochrome banding. Arrows indicate CMA^+^/DAPI° location on the long arm, L, and on the short arm, S.

Mitotic analyses of *C. crocoides* showed that all accessions are diploids (2*n* = 14). Presence of cytotypes was not observed for this species. Mitotic metaphase chromosomes and haploid idiogram are shown in Figure 4, and measurement data and karyological parameters are given in Table 6. The species presented a reasonably bimodal karyotype, with two pairs of large chromosomes and five progressively smaller ones. In this study, the haploid karyotype formula established according to the classification of Levan *et al.* (1964) was 5m (1sat) + 2sm. Total length of the haploid chromosome set (THL) was 30.90 μm, and the average chromosome length of each pair ranged from 3.50 (pair VII) to 6.72 μm (pair I). With respect to the karyotype classification of Stebbins (1971), *C. crocoides* was included in category 2A. Analyses of intrachromosomal (M_CA_) and interchromosomal (CV_CL_) karyotype asymmetry, however, showed values of 22.81 and 26.62, respectively, corresponding to relatively low karyotype asymmetry (Figure 4). In relation to heteromorphism, only pair I presented a significant difference in total length between homologues (6.41 and 7.02 μm; p = 0.035). All pairs of chromosomes presented pericentromeric regions with AT-rich DNA (CMA^°^/DAPI^+^). Clusters of GC-rich DNA (CMA^+^/DAPI^°^) were observed on chromosomes 13 and 14 (pair VII), comprising the secondary constriction. Nonetheless, most accessions presented CMA^+^ bands on the short arm of both chromosomes, except ESC 688 which presented such on the short arm of one chromosome and on the long of another (Figure 4).

**Table 6:** Karyotype features of *Calydorea crocoides*. Mean values of total chromosome length (*TCL*), long arm (S), short arm (L), and arm ratio (*r*). Chromosome type (m: metacentric; sm: submetacentric) total length of the haploid chromosome set (*THL*), mean centromeric asymmetry (*M*_CA_), coefficient of variation of chromosome length (*CV*_CL_) and karyotypic formula.

Indices	I	II	III	IV	V	VI	VII
*TCL* (μm)	6.72	5.24	4.22	3.95	3.74	3.54	3.50
*S* (μm)	3.07	1.66	1.54	1.54	1.42	1.40	1.38
*L* (μm)	3.65	3.58	2.68	2.41	2.31	2.13	2.12
*r*	1.19	2.16	1.74	1.56	1.63	1.52	1.53
Chromosome type	m	sm	sm	M	M	m	m
*THL* (μm)	30.90						
*M*_CA_	22.81						
*CV*_CL_	26.62						
Haploid karyotypic formula	5m(1sat) + 2sm						

### Morphology and stainability of pollen grains

**Figure 5:**
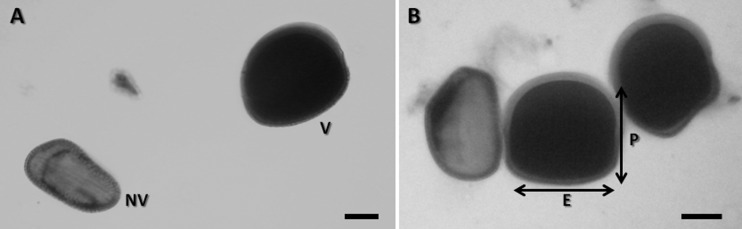
Pollen grains of *Calydorea crocoides* (population ESC692). A) Viable (*V*) and non-viable (*NV*) pollen grains; B Pollen grain measurements (P: polar axis; E: equatorial diameter). Scale bar = 10 μm.

Regarding pollen grain stainability, the three analyzed accessions (i.e., populations) showed values above 90% (Table 7). Measures of pollen grains were also performed (Figure 5). The ratio between pollen axes (P/E) ranged from 0.90 to 1.06 (Table 7), comprising pollen grains of the type prolate spheroidal and oblate spheroidal according to the classification of Erdtman (1971), both types belonging to the subesferoidal class. The pollen-type oblate spheroidal was the most common.

**Table 7:** Morphology and pollen fertility in populations of *Calydorea crocoides*. The estimated measures were polar axis (*P*), equatorial diameter (*E*), ratio between polar axes (*P*/*E*) and percentage of viability of pollen grains (%).

Population	*P* (μm)	E (μm)	P/E	Morphology	Viable pollen	Non-viable pollen	%
ESC 692	40.63^a^	38.28^a^	1.06	Prolate spheroidal	3500	356	90.76
ESC 688	39.32^b^	41.88^b^	0.94	Oblate spheroidal	3500	235	93.70
ESC 677	37.14^c^	41.20^b^	0.90	Oblate spheroidal	3500	114	96.84

Averages followed by the same letter do not differ statistically by Tukey test (p = 0.05).

### Mapping and niche modelling of the populations

**Figure 6:**
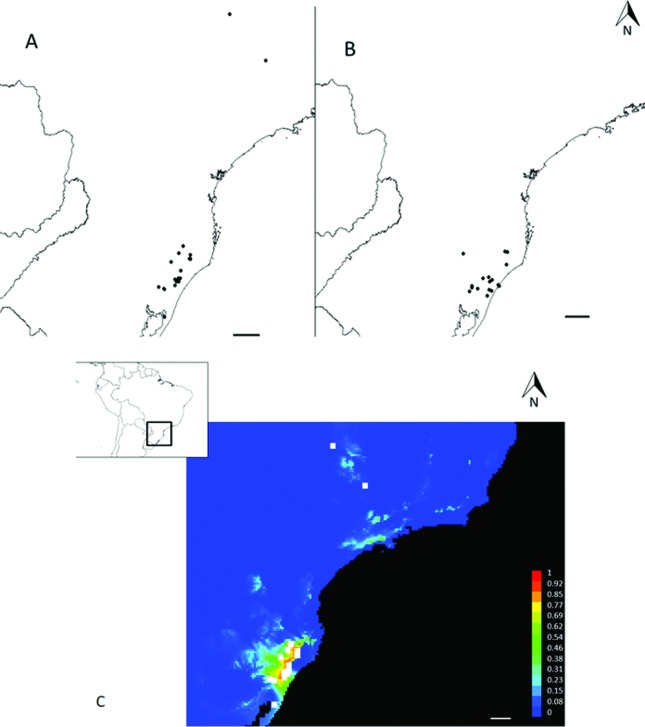
Map of the distribution of *Calydorea crocoides* (A); conservation areas (B), and potential geographical distribution for *Calydorea crocoides* according to maximum entropy (C). Similarity values the lowest training threshold are shown from light blue to red, values below this threshold are present in dark blue. Black circles indicate the accessions of *Calydorea crocoides* (A) and conservations areas (B). White squares indicate the accessions of *Calydorea crocoides* (C). Scale = 100 km.

During 12 years of collection of Iridaceae species and searches in herbaria, 39 accessions of *C. crocoides* were identified (Figure 6A). The species occurs mainly in the Campos de Cima da Serra and surroundings areas. There are records of only two collections in the state Minas Gerais. After a search to identify the existence of conservation units in these locations, 17 national and state conservation areas were found in southern Brazil (Figure 6B). No conservation areas in the collection sites of Minas Gerais were found. The model generated by MaxEnt based on all points of occurrence of *C. crocoides* showed a reduced area with suitable habitats, highlighting a narrow possibility of occurrence in other locations (Figure 6C). Suitability values of MaxEnt in the environmental space ranged 0–0.999 (± 0.001) across the study area.

## Discussion

*Calydorea crocoides* is a well-recognized species forming a robust monophyletic group with *C. campestris* (Klatt) Baker, *C. basaltica* Ravenna, *C. longipes* Ravenna and *C. approximata* R.C. Foster (Chauveau *et al.*, 2012). The species is closely related to *C. campestris*, moreover both species are morphologically very similar and their genome sizes are alike, differing from the other *Calydorea* species (Moraes *et al.*, 2015). In addition, both species present the conserved basic chromosome number of *x* = 7 and a bimodal karyotype with two long and five short chromosomes. Interestingly, in spite of all these common traits and evolutionary relationships, *C. crocoides* and *C. campestris* differ in their geographic distribution, once *C. crocoides* is very restricted and *C. campestris* occur in almost all of southern South America (Eggers, 2015). In this context, an important evolutionary question arises concerning the factors that delimit the geographic distribution of *C. crocoides*. Thus, we sought for genetic and niche modeling data as a first approach to answer this question.

### Genetic diversity and population genetic structure

In this study we obtained the first evidence concerning genetic divergence for natural populations of *C. crocoides*. These data allowed an assessment of current levels of genetic variation and population structure that may contribute to the conservation and management of such a restricted distributed species. As revealed by ISSR, *C. crocoides* has a relatively high genetic diversity at the species level (Table 3) compared to other herbaceous or endemic species (Hamrick and Godt 1990; Nybom and Bartish, 2000; Nybom, 2004). High levels of genetic diversity were also detected for other native species from South American grasslands (Jakob *et al.*, 2009; Lorenz-Lemke *et al.*, 2006, 2010; Sede *et al.*, 2012; Fregonezi *et al.*, 2013; Turchetto *et al.*, 2014; 2016; Barros *et al.*, 2015; Stiehl-Alves *et al.*, 2017), some of these being species with restricted geographical distribution (Lorenz-Lemke *et al.*, 2006, 2010). At the population level, AMOVA (Table 4) showed that most of the genetic variation was found within populations, suggesting a genetic structure characteristic of outcrossing plants (Nybom and Bartish, 2000; Nybom, 2004). Estimates of genetic diversity within populations presented here were lower than estimates for other species of Iridaceae found in South American grasslands (Tacuatiá *et al.*, 2012a; Stiehl-Alves *et al.*, 2017). Genetic diversity of plant species can be affected by many factors, such as amplitude range, duration of life cycle, mating system, and seed dispersion capacity (Hamrick and Godt, 1996; Nybom and Bartish, 2000; Nybom, 2004). These Iridaceae have some characteristics in common, such as seed dispersal by gravity (i.e., the seeds are dispersed near to the parental) and being probably long lived perennials. In addition, outcrossing was established for species of Iridaceae, and usually cross-pollinated species retain higher levels of genetic polymorphism (Hannan and Orick, 2000; Wróblewska *et al.*, 2003; Wang *et al.*, 2009; Tacuatiá *et al.*, 2012a; Stiehl-Alves *et al.*, 2017). In turn, species with a narrow geographic range, being adapted to a restricted niche, as well as endangered species, tend to maintain lower levels of genetic variation than species with broad geographic distribution (Hamrick and Godt, 1996; Hannan and Orick, 2000; Nybom and Bartish, 2000; Nybom, 2004; Szczecinska *et al.*, 2016), but exceptions may occur (Lorenz-Lemke *et al.*, 2006, 2010; Sede *et al.*, 2012; Turchetto *et al.*, 2016).

Although the global *F*_ST_ values point to more structured populations, which is consistent with other species of Iridaceae (Hannan and Orick, 2000; Wróblewska *et al.*, 2003; Wang *et al.*, 2009; Tacuatiá *et al.*, 2012a; Stiehl-Alves *et al.*, 2016, 2017), the pairwise comparison of *F*_ST_ revealed that some populations are less differentiated from each other (*F*_ST_ < 0.20) and retain residual gene flow among them (*N*_*m*_ > 1) (Table 5). The population genetic structure in plants reflects the interaction of many evolutionary processes, including habitat fragmentation and population isolation, mutation, genetic drift, mating system, gene flow, and selection (Hamrick and Godt, 1996; Hannan and Orick, 2000; Nybom and Bartish, 2000; Nybom, 2004; Stiehl-Alves *et al.*, 2016). In this sense, although the STRUCTURE analysis detected two genetic clusters for the molecular dataset (Figure 2), consistent with the UPGMA dendrogram (Figure 3), the estimates of pairwise gene flow support the result of the STRUCTURAMA analysis (*K* = 1), since gene flow was detected even for the most differentiated populations (ESC218 and ESC661). However, considering that *C. crocoides* is a geophyte, part of the estimated gene flow may be a reflection of an extensive genetic connection between populations in the past. Currently, southern Brazilian grasslands have been highlighted as threatened by habitat loss and fragmentation, due to severe and long-term human disturbance, and studies have highlighted the need to implement conservation strategies (Lorenz-Lemke, 2006, 2010; Iganci *et al.*, 2011; Fregonezi *et al.*, 2013; Barros *et al.*, 2015).

### Cytogenetic characterization

Confirming previous data of our research team (Moraes *et al.*, 2015), all the populations of *C.* c*rocoides* analyzed are diploids with 2*n* = 14. The basic chromosome number *x* = 7 plus some morphological characters are considered synapomorphies for Tigridieae (Goldblatt, 1982). Although polyploid species are known for *Calydorea* and other Iridaceae species (Goldblatt, 1982; Goldblatt and Takei, 1997; Souza-Chies *et al.*, 2012; Moraes *et al.*, 2015), polyploidy was not observed in *C. crocoides*.

Bimodality is considered a conserved character for Tigridieae and reinforces the monophyly of the tribe (Goldblatt, 1982; Kenton *et* al., 1990; Moraes *et al.*, 2015). Our data showed that *C. crocoides* keeps this conserved feature even though chromosomes of the smaller set decrease gradually in size from the largest to the smallest ones, making this character less marked than in other Tigridieae (Goldblatt, 1982; Kenton and Heywood, 1984; Alves *et al.*, 2011; Moraes *et al.*, 2015). Furthermore, the karyotype of *C. crocoides* is rather low asymmetric, contrasting with some species of Tigridieae already evaluated in terms of intra- and interchromosomal asymmetries and Stebbins’ classification (Alves *et al.*, 2011). The evaluation of karyotypic symmetry is of extreme relevance for inferences about chromosomal evolution, especially for Iridaceae in which chromosomal rearrangements, dysploidy and polyploidy result in a great diversity concerning chromosome number and size (Kenton *et al.*, 1990; Goldblatt and Takei, 1997). According to Stebbins (1971), the analysis of karyotype symmetry together with morphological features allows to determine the evolutionary direction, once there is a tendency to evolve from symmetrical karyotypes towards asymmetry in derived species. In this sense, the less asymmetric karyotype of *C. crocoides* could suggest a more ancestral position in the genus. Nevertheless, *Calydorea* is not monophyletic and its species are split into some lineages that group with other genera (Chauveau *et al.*, 2012). Taking this into account, as well as the conservation of karyotypic features in Tigridieae, an analysis of additional cytological markers through *in situ* hybridization for example, is necessary to better understand the evolutionary relationships within this genus.

Chromosomes in *C. crocoides* are of metacentric and submetacentric type, different from what was suggested in a previous study based on meiotic analysis, in which, besides metacentric and submetacentric types, one pair of telocentric chromosomes was suspected (Moraes *et al.*, 2015). A population difference could, however, be considered in this case. Chromosome heteromorphisms are frequent in Tigridieae, with records for *Eleutherine bulbosa*, *Gelasine elongata* and *Cipura paludosa* (Kenton and Rudall, 1987; Guerra, 1988, 1991; Alves *et al.*, 2011). Such heteromorphisms resulted of inversions followed by tandem duplication or translocations, and are important to the speciation process in this plant group (Moraes *et al.*, 2015). In this study, CMA/DAPI chromosome banding of *C. crocoides* was evaluated for the first time. All plants assessed presented the same pattern of DAPI^+^ pericentromeric signals on the 14 chromosomes. A CMA^+^ band was seen on the short arms of both chromosomes of pair VII in plants from the ESC 684, ESC 692, ETLP 807 and ETLP 812 populations, while ESC 688 presented heterozygosity in this pair, indicating a pericentric inversion. Assuming that pollen stainability indicates viability, the observed chromosome variation apparently did not affect male fertility, since this accession presented a higher index of viable grains than the other analyzed accessions. For *Eleutherine bulbosa,* a heterozygosity in pair I was previously reported, although the meiotic behavior is normal and asexual reproduction ensures the maintenance of chromosome heterozygosity. Taking into account the heteromorphism in chromosome pair I, we may not rule out the possibility of translocation events, but, more cytogenetic markers should be analyzed to determine exactly which chromosome rearrangements have occurred.

### Morphology and pollen viability

The quantity and quality of pollen produced by a flower is an important component of its fitness (Dafni and Firmage, 2000; Kelly *et al.*, 2002), with quality often being equated with pollen viability (Stanley and Linskens, 1974; Heslop-Harrison *et al.*, 1984). A common method to evaluate the amount of pollen and its viability is staining and direct counting (Alexander, 1980; Barrett, 1985; Dudash, 1991). In all populations analyzed for *C. crocoides*, without exception, the average of viable pollen grains was significantly higher than that of unviable ones (Table 7). High rates of pollen viability may be an indication that the plants are male-fertile. Although meiotic behavior was not analyzed, the high pollen viability (90.73% - 96.84%) suggests a regular meiotic process. The individuals of *C. crocoides* analyzed by Moraes *et al.* (2015) presented high pollen viability, which corroborated the meiotic stability data. In a study based on pollen morphology in Tigridieae, Rudall and Wheeler (1988) found that form and size of the pollen grains can differ considerably, with size varying between and within species. According to these authors, pollen variability within a single species is not uncommon. This variation is well represented in our results, since two different morphologies could be observed among the analyzed specimens, as well as size differences.

### Implications for conservation

The success of the maintenance and preservation of a species depends on a good understanding of the level and distribution of genetic variation (Toro and Caballero, 2005; Frankham *et al.*, 2010; Furlan *et al.*, 2012; Kahilainena *et al.*, 2014; Pérez *et al.*, 2015). Genetic diversity in natural populations may significantly affect the survival and evolution of species or populations in changing environments, and therefore, any conservation effort should aim to preserve a maximum of genetic diversity within the pool of target genes (Toro and Caballero, 2005). Habitat fragmentation, predominantly attributed to anthropogenic pressures causing a reduction in the population size, makes a species susceptible to loss of genetic polymorphism by the effect of random genetic drift and inbreeding (Frankham *et al.*, 2010; Furlan *et al.*, 2012). At the species level, *C. crocoides* presents high genetic diversity, and the populations analyzed here have singular cytogenetic features that deserve to be maintained. Niche modeling also demonstrated few suitable habitats for *C. crocoides*. In spite of some populations being found within protected areas in Subtropical Highland Grasslands (Aparados da Serra National Park), this region is nowadays threatened by antropogenic activities, such as the introduction of alien species, or over-use by fire and cattle grazing (Overbeck *et al.*, 2007; Iganci *et al.*, 2011; Barros *et al.*, 2015). Thus, it is necessary to establish conservation strategies for *C. crocoides* in order to maintain populations with unique characteristics. Reviewing the threat status indicated by the IUCN red list is one of the necessary procedures. Here, we suggest a change from the Least Concern to the Vulnerable category, consistent with the status actually established by the regional list published by the Rio Grande do Sul state government.

## Conclusions

The results reported in this study showed that the surveyed populations of *Calydorea crocoides* have singular characteristics that emphasized that planning is necessary for *in situ* maintenance of this species. For instance, the cytological data highlighted the presence of a chromosomal rearrangement exclusive to population ESC688. This emphasizes the importance of collecting plants from different populations in plant cytogenetic investigations, primarily when searching for genetic polymorphisms, in a manner to contribute to diversity studies. Our analysis also highlighted that the morphology of pollen grains is variable and pollen viability is high in the analyzed populations. The surveyed populations presented high levels of genetic diversity. Although low genetic diversity is common in species with restricted distribution, some studies have reported cases of endemic species where genetic variation is high. Our niche modeling approach confirmed narrow range of sites suitable for populations of *C. crocoides*. In this context, our data allow to suggest changes from the “least concern” (LC) to the “vulnerable” (VU) status in the IUCN Red List, especially considering that the species is threatened by current or potential levels of exploitation of habitat and by the effects of introduced taxa. Our data provide an glimpse into the variability and evolution of *C. crocoides* that can serve as a starting point for further biological and evolutionary studies. Along this perspective, we intend to analyze the main factors involved in the dispersion power in *C. crocoides* and to compare it with *C. campestris* that presents a wider geographic distribution.

## Supplementary material

The following online material is available for this article:

Table S1 - Collection sites of *Calydorea crocoides* and conservations units.

Table S2 - Pairwise Nei’s genetic identity for *Calydorea crocoides*.
