# Supplementary material for: Genetic and ecological niche modeling of Calydorea crocoides (Iridaceae): an endemic species of Subtropical Highland Grasslands
Source: Genet Mol Biol. 2018;41(1 Suppl 1):327–40. doi: 10.1590/1678-4685-GMB-2017-0078 (PMC5913728; doi:10.1590/1678-4685-GMB-2017-0078)
Supplement: Supplementary file 1 [file 1415-4757-GMB-41-01-2017-0078-s001.pdf]

Supplementary material to "Genetic and ecological niche modeling of *Calydorea crocoides* (Iridaceae): an endemic species of Subtropical Highland Grasslands"

Table S1 – Data of the collection sites of *Calydorea crocoides* and conservations units used for the mapping.

**Accessions**	**Latitude (°S)**	**Longitude (°W)**	**State**	**City**
ICN 173563	28°10'19.90"S	50°00'50.70"W	Santa Catarina	São Joaquim
ICN 173564	29°04'04.90"S	50°02'40.20"W	Rio Grande do Sul	Cambará do Sul
ICN 173565	29°26'32.80"S	50°36'38.90"W	Rio Grande do Sul	São Francisco de Paula
ICN 173566	29°27'00.50"S	50°36'14.10"W	Rio Grande do Sul	São Francisco de Paula
ICN 173567	28°48'09.20"S	49°57'03.50"W	Rio Grande do Sul	São José dos Ausentes
ICN 173568	29°26'30.40"S	50°36'38.78"W	Rio Grande do Sul	São Francisco de Paula
ICN 173569	28°21'41.00"S	49°35'20.00"W	Santa Catarina	Bom Jardim da Serra
ICN 180198	29°18'59.80"S	50°09'59.50"W	Rio Grande do Sul	São Francisco de Paula
ICN 180248	28°28'53.90"S	50°19'49.00"W	Rio Grande do Sul	Bom Jesus
ICN 180199	28°13'43.20"S	49°50'51.30"W	Santa Catarina	São Joaquim
ICN 180200	28°23'08.30"S	49°33'18.60"W	Santa Catarina	Bom Jardim da Serra
ICN 173572	29°09'44.50"S	50°05'40.20"W	Rio Grande do Sul	Cambará do Sul
ICN 173589	29°27'24.20"S	50°36'32.60"W	Rio Grande do Sul	São Francisco de Paula
ICN 173570	29°06'27.54"S	50°10'03.79"W	Rio Grande do Sul	Cambará do Sul
ICN 173571	29°10'31.00"S	50°07'0.00"W	Rio Grande do Sul	Cambará do Sul
ICN 173588	29°10'31.30"S	50°07'0.30"W	Rio Grande do Sul	Cambará do Sul
ICN 173570	29°06'28.00"S	50°04'04.00"O	Rio Grande do Sul	Cambará do Sul

Accessions	Latitude (°S)	Longitude (°W)	State	City
ICN 173574	29°03'47.00"S	49°57'21.00"W	Rio Grande do Sul	Cambará do Sul
ICN 173575	29°10'43.60"S	50°01'35.00"W	Santa Catarina	Praia Grande
ICN 173569	28°21'41.00"S	49°35'20.00"W	Santa Catarina	Bom Jardim da Serra
ICN 180202	29°26'51.00"S	50°36'18.00"W	Rio Grande do Sul	São Francisco de Paula
ICN 180201	30°26'32.00"S	50°36'38.00"W	Rio Grande do Sul	São Francisco de Paula
ICN 180203	29°27'16.00"S	50°36'25.00"W	Rio Grande do Sul	São Francisco de Paula
NY00527607	29°11'03.32"S	50°00'53.55"W	Santa Catarina	Praia Grande
MBM0172196	29°11'03.32"S	50°00'53.55"W	Santa Catarina	Praia Grande
Z000099049	29°11'03.32"S	50°00'53.55"W	Santa Catarina	Praia Grande
ICN 026300	29°22'42.50"S	50°49'27.82"W	Rio Grande do Sul	Canela
CTES0561463 60382	28°22'19.16"S	49°33'50.88"W	Santa Catarina	Bom Jardim da Serra
UEC096407 143265	28°21'57.18"S	49°34'45.66"W	Santa Catarina	Bom Jardim da Serra
UEC056178 76999	28°23'33.75"S	49°32'58.60"W	Santa Catarina	Bom Jardim da Serra
MBM0386979	27°55'39.00"S	49°51'19.00"W	Santa Catarina	Urupema
RB00925645 617170	27°55'39.00"S	49°51'19.00"W	Santa Catarina	Urupema
HUEFS 210763	27°55'39.00"S	49°51'19.00"W	Santa Catarina	Urupema
MBM0303287	21°21'22.17"S	46°31'47.32"W	Minas Gerais	Moçambinho
PACA-AGP 3435a	19°42'54.55"S	47°58'51.84"W	Minas Gerais	Uberaba
Conservation Units				
Parque Nacional de São Joaquim	28°8'4"S	49°28'47"W	Santa Catarina	Urubici
Reserva Biológica Aguaí	28°35'56"S	49°25'31"W	Santa Catarina	Siderópolis
Parque Estadual da Serra Furada	28° 9' 23"S	49°23'32"W	Santa Catarina	Orleans
Parque Nacional de Aparados da Serra	29°11'30"S	50°5'51"W	Rio Grande do Sul	Cambará do Sul
Floresta Nacional de São Francisco de Paula	29°25'22"S	50°23'11"W	Rio Grande do Sul	São Francisco de Paula
Floresta Nacional de Canela	29°21'42"S	50°48'46"W	Rio Grande do Sul	Canela
Estação Ecológica de Aracuri-Esmeralda	28°13'31"S	51°9'57"W	Rio Grande do Sul	Muitos Capões

Accessions	Latitude (°S)	Longitude (°W)	State	City
Parque Nacional da Serra Geral	29°8'2"S	49°59'40"W	Rio Grande do Sul	Cambará do Sul
Refúgio de Vida Silvestre da Ilha dos Lobos	29°20'49"S	49°42'16"W	Rio Grande do Sul	Torres
Parque da Guarita	29°21'16"S	49°43'59"W	Rio Grande do Sul	Torres
Parque do Caracol	29°21'42"S	50°48'46"W	Rio Grande do Sul	Canela
Parque Estadual do Ibitiriá	29°32'30"	50°54'51"W	Rio Grande do Sul	Bom Jesus
Parque Estadual do Tainhas	29°5'4.58"S	50°21'57.6"W	Rio Grande do Sul	São Francisco de Paula
Área de Proteção Ambiental Rota do Sol	29º02'52"S	50º08'41"W	Rio Grande do Sul	Cambará do Sul
Área de Proteção Ambiental Rota do Sol	29°29'20"S	50°6'49"W	Rio Grande do Sul	Itati
Área de Proteção Ambiental Rota do Sol	29°42'16"S	50°11'46"W	Rio Grande do Sul	Maquiné
Área de Proteção Ambiental Rota do Sol	29°26′49″S	50°34′45″W	Rio Grande do Sul	São Francisco de Paula
Área de Proteção Ambiental Rota do Sol	29°32'12"S	50°1'44"W	Rio Grande do Sul	Três Forquilhas
Estação Ecológica Estadual Aratinga	29°26′49″S	50°34′45″W	Rio Grande do Sul	São Francisco de Paula
Estação Ecológica Estadual Aratinga	29°29'20"S	50°6'49"W	Rio Grande do Sul	Itati
Reserva Biológica Estadual Mata Paludosa	29°29'20"S	50°6'49"W	Rio Grande do Sul	Itati
Parque Estadual de Itapeva	29°19'27"S	49°45'28"W	Rio Grande do Sul	Torres
