# Supplementary material for: Genetic and ecological niche modeling of Calydorea crocoides (Iridaceae): an endemic species of Subtropical Highland Grasslands
Source: Genet Mol Biol. 2018;41(1 Suppl 1):327–40. doi: 10.1590/1678-4685-GMB-2017-0078 (PMC5913728; doi:10.1590/1678-4685-GMB-2017-0078)
Supplement: Supplementary file 2 [file 1415-4757-GMB-41-01-2017-0078-s002.pdf]

Genetics and Molecular Biology

Supplementary material to "Genetic and ecological niche modeling of *Calydorea crocoides* (Iridaceae): an endemic species of Subtropical Highland Grasslands"

Table S2 - Pairwise Nei's genetic identity estimated for populations of *Calydorea crocoides*.

**Populations**								
	ESC661	ESC218	ESC677	ESC684	ESC688	ESC692	JLM01	DR71
ESC661	1.000							
ESC218	0.866	1.000						
ESC677	0.855	0.813	1.000					
ESC684	0.854	0.857	0.898	1.000				
ESC688	0.889	0.856	0.905	0.886	1.000			
ESC692	0.827	0.815	0.816	0.777	0.821	1.000		
JLM01	0.856	0.830	0.882	0.860	0.892	0.864	1.000	
DR71	0.832	0.826	0.892	0.884	0.900	0.769	0.880	1.000
